# Studies on the synthesis and stability of *α*-ketoacyl peptides

**DOI:** 10.1007/s00726-020-02902-8

**Published:** 2020-10-14

**Authors:** Johann Sajapin, Michael Hellwig

**Affiliations:** 1 Chair of Food Chemistry, Technische Universität Dresden, 01062 Dresden, Germany; 2 Institute of Food Chemistry, Technische Universität Braunschweig, Schleinitzstraße 20, 38106 Braunschweig, Germany

**Keywords:** Protein oxidation, Backbone cleavage, Ketoacyl peptide, Schiff base, Transamination

## Abstract

Oxidative stress, an excess of reactive oxygen species (ROS), may lead to oxidative post-translational modifications of proteins resulting in the cleavage of the peptide backbone, known as *α*-amidation, and formation of fragments such as peptide amides and *α*-ketoacyl peptides (*α*-KaP). In this study, we first compared different approaches for the synthesis of different model *α*-KaP and then investigated their stability compared to the corresponding unmodified peptides. The stability of peptides was studied at room temperature or at temperatures relevant for food processing (100 °C for cooking and 150 °C as a simulation of roasting) in water, in 1% (*m*/*v*) acetic acid or as the dry substance (to simulate the thermal treatment of dehydration processes) by HPLC analysis. Oxidation of peptides by 2,5-di-*tert*-butyl-1,4-benzoquinone (DTBBQ) proved to be the most suited method for synthesis of *α*-KaPs. The acyl side chain of the carbonyl-terminal α-keto acid has a crucial impact on the stability of *α*-KaPs. This carbonyl group has a catalytic effect on the hydrolysis of the neighboring peptide bond, leading to the release of *α*-keto acids. Unmodified peptides were significantly more stable than the corresponding *α*-KaPs. The possibility of further degradation reactions was shown by the formation of Schiff bases from glyoxylic or pyruvic acids with glycine and proven through detection of transamination products and Strecker aldehydes of *α*-keto acids by HPLC–MS/MS. We propose here a mechanism for the decomposition of *α*-ketoacyl peptides.

## Introduction

Aerobic cellular respiration is common for all known animals. Though highly energetically beneficial, it still bears some risks, since it makes the exposure of the organism to reactive oxygen species (ROS) inevitable. For example, ROS have been shown to be formed in vivo in mitochondria as a byproduct of glucose metabolism due to leakage of electrons from the transport chain (Turrens and Boveris 1980) or in activated phagocytic cells as a response to microorganisms (Curnutte and Babior 1987). These reactive compounds have the potential to inflict severe damage on cells, so aerobic organisms use an abundance of protective endogenous antioxidant systems to maintain a precise balance between the formation and neutralization of ROS. In the case of insufficient protective mechanisms (for example, under-expression of superoxide dismutase (Schwartz et al. 1998)) or an excess of ROS (e.g., through influx with cigarette smoke; Valavanidis et al. 2009) the balance can be disrupted, so diverse biomolecules may be oxidized. This process is generally described as oxidative stress, and since proteins make up to half of dry cell mass, they are likely to become a major subject of an oxidative attack resulting in a post-translational modification (Esterbauer et al. 1992; Davies 2005).

These reactions were predominantly studied regarding their impact on human metabolism and health (Schöneich et al. 2003; Dean et al. 1997). More recent studies examine the susceptibility of food proteins to oxidation in the course of processing and storage, as well as the consequences on food quality and digestibility (Feng et al. 2015; Utrera and Estévez 2013; Sante-Lhoutellier et al. 2007). Though many studies predominantly focus on specific oxidation markers and products, elucidation of reaction pathways was sought after as well. Very often, protein oxidation in food is assessed by quantification of protein carbonyls, which comprise a large set of individual structures among which glutamic and aminoadipic semialdehydes predominate (Havelund et al. 2017; Hellwig 2019).

**Fig. 1:**
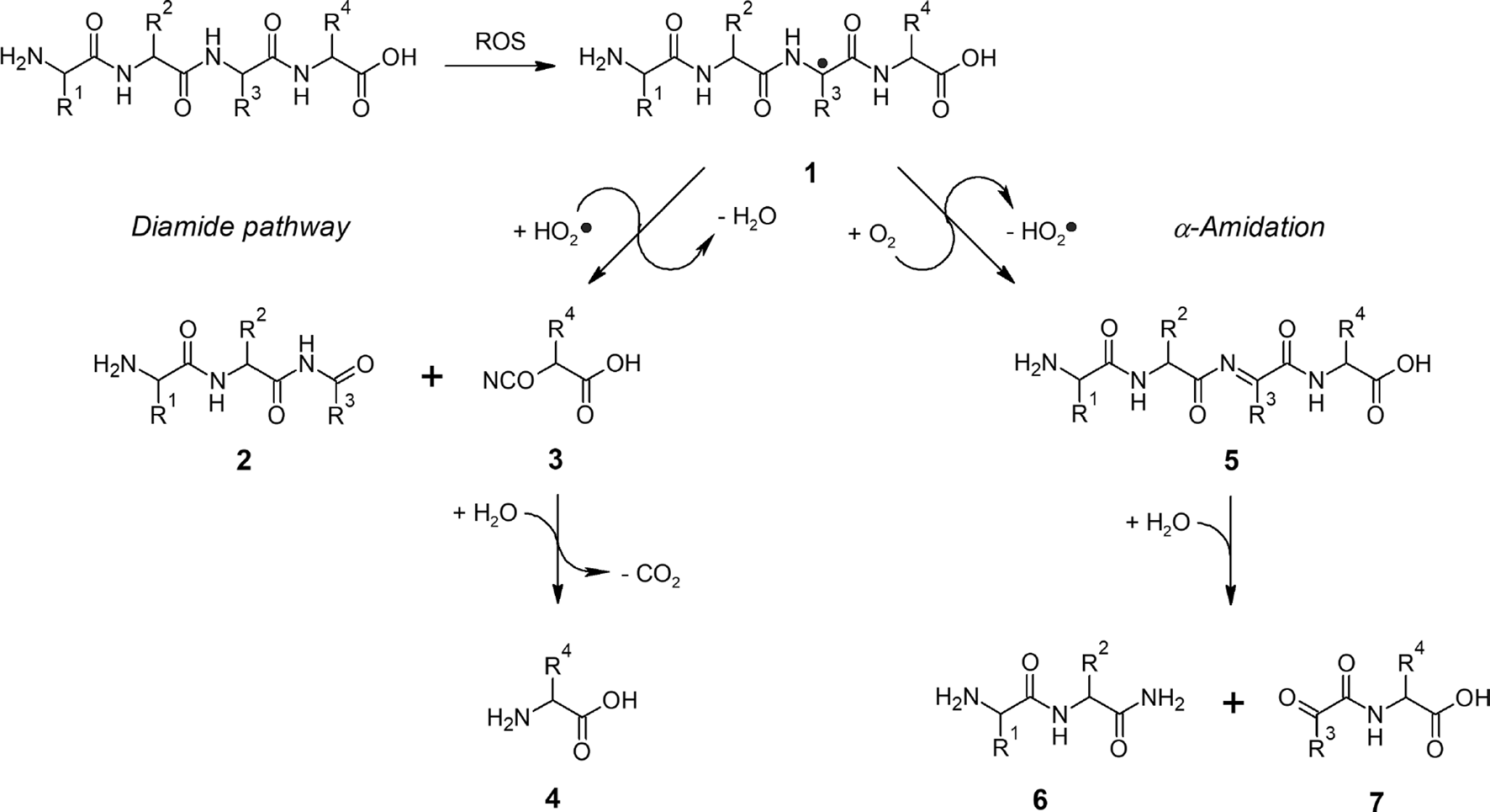
Mechanism of peptide backbone cleavage, initiated by attack of ROS on a peptide, abstraction of the *α*-C hydrogen atom, creation of a carbon-centered radical (1) and rapid decomposition via the diamide pathway or *α*-amidation. The former leads to the formation of an *N*-terminal carbonyl compound (2) and a C-terminal isocyanate (3), which can further react with water to an amino compound (4). The *α*-amidation leads to an unsaturated peptide species (5), further decomposing into a peptide amide (6) and an *α*-ketoacyl peptide (7). R^1^, R^2^, R^3^, R^4^ are amino acid side chains

In general, the post-translational oxidative modification of proteins is represented by modification on side chains of amino acids (Stadtman 1993), cross-linking (Mirzaei and Regnier 2006) and fragmentation (Davies 1996). Especially oxidative fragmentation reactions of proteins under the conditions of food processing need to be explored in more detail. Based on previous studies (Garrison 1987; Garrison et al. 1962, 1970; Stadtman 2006), a simplified peptide backbone cleavage mechanism can be described, as shown in Fig. 1. The process is initiated by an attack of ROS on the polypeptide and the subsequent abstraction of an *α*-C hydrogen, leading to the formation of a carbon-centered peptide radical (1), stabilized by resonance structures of both neighboring peptide groups. In the presence of oxygen, this compound becomes subject of conversion including several highly reactive species such as peptide peroxyl and peptide alkoxyl radicals, which can decompose via the diamide pathway or *α*-amidation, the former leading to the formation of an imide at the N-terminal truncated peptide (2) and an isocyanate at the C-terminal peptide (3), which can further react with water to restore the amino acid structure (4). The *α*-amidation leads to an unsaturated peptide (5), further decomposing into a peptide amide (6) and an *α*-ketoacyl peptide (7).

Some recent studies indicate that certain amino acid side chains (especially that of methionine) are not only prone to oxidation by reactive species but can further transfer the oxidative damage to the peptide backbone. The subsequently accelerated formation of a carbon-centered radical and the following *α*-amidation lead to higher yields of peptide amides (Headlam et al. 2000; Hellwig et al. 2015) and α-ketoacyl peptides. Some individual peptide amides are known to be bioactive and participate in the nervous and endocrine systems (Grimmelikhuijzen and Graff 1986; Chufan et al. 2009). The *α*-ketoacyl peptides are predominantly considered as a part of the protein carbonylation reaction (Utrera and Estévez 2012), so the reactivity of these compounds remains to be explored.

In the present study, we, therefore, compared different synthetic approaches to *α*-ketoacyl peptides regarding their complexity and feasibility. Five *α*-ketoacyl peptides were synthesized in sufficient amounts to allow for a study on their reactivity. For the first time, the stability of model *α*-ketoacyl peptides with each other and corresponding unmodified peptides was compared, discovering a crucial effect of the *α*-carbonyl group. A new catalytic mechanism is proposed. We further identified and quantified the decomposition products of *α*-ketoacyl peptides and propose a combined pathway of advanced decomposition reactions.

## Materials and methods

### Chemicals

Pyruvic acid and silica gel 60 were obtained from Acros Organics (Geel, Belgium), and 5-(dimethylamino)-naphthalene-1-sulfonyl chloride (dansyl chloride), pyridine, glycine, and 2,5-di-*tert*-butyl-1,4-benzoquinone (DTBBQ) from Alfa Aesar (Kandel, Germany). Glycylalanine (H-GA-OH), and Boc-Isoleucylglycine (Boc-IG-OH), were purchased from Bachem (Bubendorf, Switzerland). Ethyl acetate from BCD Chemie (Hamburg, Germany), ethanol from Berkel Alkoholhandel (Berlin, Germany) and acetic acid for synthesis, and formic acid for HPLC from Carl Roth (Karlsruhe, Germany) were used. Acetonitrile LC–MS grade and *n*-hexane were from Th. Geyer (Renningen, Germany). Trifluoroacetic acid (for HPLC) was obtained from Fisher Scientific (Loughborough, UK), glyoxylic acid monohydrate and acetaldehyde from Fluka Chemicals (Buchs, Switzerland). 1-Butanol, potassium iodide, ammonium formate, cupric sulfate pentahydrate, sodium carbonate, sodium hydrogen carbonate and sodium hydroxide were purchased from Grüssing (Filsum, Germany), and *o*-dianisidine from Lancaster Synthesis (Morecambe, UK). Acetone, 2,4-dinitrophenyl hydrazine (DNPH), deuterium oxide, alanylglycine (H-AG-OH), nonafluoropentanoic acid (NFPA), hydrochloric acid, glycylglycine, and glycylglycylglycine (H-GGG-OH) were from Sigma-Aldrich (Steinheim, Germany). Sodium sulfate (anhydrous), 4-methylbenzenesulfonylchloride (tosyl chloride), acetonitrile (HPLC grade), methanol and tetrahydrofuran were obtained from VWR (Darmstadt, Germany). 2,2-Dihydroxy-1*H*-indene-1,3(2*H*)-dione (ninhydrin) was from Serva Feinbiochemica (Heidelberg, Germany). Water for solutions, buffers, and HPLC eluents was obtained from a Bi 18 E double distillation system (QCS, Maintal, Germany).

### Model incubations

Each substance was incubated as 1 mM solution in water or in 1% (*m*/*v*) acetic acid. Unmodified peptides and *α*-ketoacyl peptides were also incubated as dry substances. For that, 1 mM solutions were divided in 1 mL fractions and separately lyophilized in glass vials. Aqueous solutions were incubated in screw-cap tubes at room temperature for up to 48 h to simulate the treatment during the marination process, and at 100 °C and 150 °C for up to 120 min to mimic the respective thermal treatment in the course of cooking and roasting in oven, then rapidly cooled on ice and refrigerated at – 32 °C. The incubations of dry substances were conducted directly in sealed glass vials at 100 °C and 150 °C to imitate the overall process of intensive dehydration under exposure to heat. The residue was rapidly cooled to room temperature, dissolved in 1 mL water and refrigerated at – 32 °C.

### Thin layer chromatography

TLC was used for quick reaction controls. Reaction mixtures (2 µL) were applied on a silica gel 60 plate (Merck, Darmstadt, Germany) and developed in a double-trough chamber. A solution of 1-butanole, glacial acetic acid and water (3/1/1, *v*/*v*/*v*) was used as the mobile phase. The developed plates were dried at 50 °C for 10 min and treated with a coloring agent. All substances containing carbonyl groups were sprayed with a 0.1% (*m*/*v*) solution of DNPH in 2 M hydrochloric acid. Yellow–orange colored spots revealed the presence of carbonyl compounds. Secondary amines and peptides were treated with chlorine gas for 30 min followed by evaporation of excessive chlorine and spraying with a saturated solution of *o*-dianisidine and 0.05 M potassium iodide in 2% (*v*/*v*) acetic acid. Primary and secondary amines appeared as black spots. Specific detection of primary amines was carried out by spraying of developed plates with a 0.1% (*m*/*v*) solution of ninhydrin in 0.05 M ethanolic acetic acid and subsequent incubation at 50 °C in a drying chamber for 10 min. Primary amines showed violet spots.

### High-performance liquid chromatography with ultraviolet detection

All high-performance liquid chromatography analyses were performed on a high-pressure gradient system, consisting of a pump P1000 with an online degasser (Knauer, Berlin, Germany), a column oven and a diode array detector Azura DAD 2.1L. Analyses were carried out using a stainless steel column (250 mm × 4.6 mm; Knauer, Berlin, Germany) filled with Eurospher-100 RP C-18 material (5 µM particle size) with a guard column (5 mm × 4 mm) filled with the same material.

#### Peptides and amino acids

The analyses of peptides were carried out after derivatization with dansyl chloride. For this, 50 µL of the sample solution was added to 75 µL of 80.8 mM lithium carbonate buffer, pH 9.5 and mixed with 100 µL of 0.5% (*m*/*v*) solution of dansyl chloride in acetonitrile for 1 h at 40 °C. The reaction was stopped afterwards by adding 10 µL of 3 M hydrochloric acid. The whole mixture was centrifuged for 5 min at 10,600*g* and the supernatant was directly used for the analysis. The injection volume was 20 µL, the separation was performed at 40 °C and at a flow rate of 1 mL/min. As mobile phases, 0.1 M formic acid in water (solvent A), and 0.1 M formic acid in a mixture of acetonitrile and water (90/10, *v*/*v*; solvent B) were used in a gradient program (0 min, 20% B; 3 min, 20% B; 11 min, 75% B; 14 min, 80% B; 16 min, 80% B; 19 min, 20% B; 24 min, 20% B). The absorbance was read at 340 nm. External calibrations were performed with peptide and amino acid solutions in a concentration range between 100 and 1300 µM under inclusion of the dansylation step.

#### Carbonyl compounds

The analyses of *α*-ketoacyl peptides and other carbonyl compounds were carried out after derivatization with DNPH. For this, 100 µL of filtered samples was mixed with 100 µL of 0.1% (*m*/*m*) DNPH solution in 2 M hydrochloric acid and incubated at room temperature for 5 min followed by addition of 300 µL acetonitrile and diluted with 500 µL of solvent A for analysis. The injection volume was 20 µL, the separation was performed at room temperature and at a flow rate of 1 mL/min. As mobile phases, 0.1% (*v*/*v*) trifluoroacetic acid in a mixture of water and acetonitrile (90/10, *v*/*v*; solvent A), and 0.1% (*v*/*v*) trifluoroacetic acid in a mixture of acetonitrile and water (90/10, *v*/*v*; solvent B) were used in a gradient program (0 min, 0% B; 10 min, 0% B; 20 min, 10% B; 60 min, 100% B; 65 min, 0% B; 75 min, 0% B). The absorbance was read at 214 and 365 nm. External calibrations were performed with peptide and amino acid solutions in a concentration range between 10 and 1200 µM.

### High-performance liquid chromatography with mass spectrometric detection

The high-pressure gradient system 1200 series (Agilent Technologies, Böblingen, Germany) consisting of a binary pump, a column oven, an autosampler, and a diode array detector, coupled with a mass spectrometer 6410 Triple Quad (Agilent) with Electrospray Ionization (ESI) was used. The mass spectrometer was run in the positive mode, with a source temperature of 350 °C and a capillary voltage of 4000 V using nitrogen (nitrogen generator 5183–2003, Agilent) as the nebulizing gas with a flow rate of 11 L/min and a pressure of 35 psi. Product ion spectra were recorded at a fragmentor voltage of 100 V and a collision energy of 10 eV. Data acquisition and evaluation were conducted with the software Mass Hunter B.02.00 (Agilent).

#### α-Ketoacyl peptides

Before analysis, 50 µL of sample taken during the incubation experiments was mixed with 450 µL of 10 mM NFPA, centrifuged at 10,600*g* for 5 min, and 250 µL of the supernatants were subjected to analysis. The injection volume was 10 µL, and the separation was performed at 35 °C and at a flow rate of 0.25 mL/min on a stainless steel column Zorbax 300 SB (50 mm × 2.1 mm; Agilent) filled with RP C-18 material (particle size 3.5 µm) using 10 mM NFPA in water (solvent A) and 10 mM NFPA in acetonitrile (solvent B) as mobile phases in a gradient program (0 min, 10% B; 15 min, 66% B; 19 min, 66% B; 20 min, 10% B; 28 min, 10% B). The absorbance was read at 230 and 280 nm, and spectra were recorded between 200 and 350 nm (increment, 1 nm). Product ion spectra were recorded with the conditions listed in Table 1.

**Table 1:** Operating conditions for recording of product ion spectra by HPLC–MS/MS

Substance	Column	Precursor ion (*m/z*)
(3-Methyl-2-oxo)-valerylglycine	Zorbax 300 SB	188
Glyoxylglycine	Zorbax 300 SB	132
Glyoxylglycylglycine	Zorbax 300 SB	189
Pyruvoylglycine	Zorbax 300 SB	146
Pyruvoylglycylalanine	Zorbax 300 SB	217
*N*-(1-Carboxyethylidene)-glycine	SeQuant ZIC-HILIC	146
*N*-(Carboxymethylidene)-glycine	SeQuant ZIC-HILIC	132

All spectra were recorded in the positive mode

#### Schiff bases

Prior to mass spectral analysis, 100 µL of a sample taken during the incubation experiments was mixed with 400 µL of HILIC solvent B, membrane filtrated (0.45 µm) and subjected to spectral analysis. The injection volume was 10 µL. The separation was performed at 35 °C and at a flow rate of 0.2 mL/min on a stainless steel column SeQuant ZIC-HILIC (100 mm × 2.1 mm, 3.5 µM, 100 Å; Merck KGaA, Darmstadt, Germany) using 5 mM ammonium formate and 0.1% (*v*/*v*) formic acid in water (HILIC solvent A) and 2 mM ammonium formate and 0.1% (*v*/*v*) formic acid in a mixture of acetonitrile and water (80/20, *v*/*v*; HILIC solvent B) in a gradient program (0 min, 95% B; 2 min, 95% B; 6 min, 85% B; 7 min, 65% B; 9 min, 65% B; 10 min, 95% B; 18 min, 95% B) as published previously (Kölpin and Hellwig 2019). Product ion spectra were recorded with the conditions listed in Table 1.

### Amino acid analysis

All samples were subjected to acid hydrolysis prior to amino acid analysis. For that, 5 mg of dry substance was mixed with 2 mL of 6 M hydrochloric acid in screw-cap tubes and incubated in a drying chamber at 110 °C for 23 h. An aliquot of 300 µL of the hydrolysates was dried afterwards in a vacuum centrifuge (SPD Speed Vac; Thermo Fischer Scientific, Karlsruhe, Germany). The dry residues were dissolved in 600 µL of loading buffer (0.12 N lithium citrate, pH 2.20), membrane filtered (0.45 µm) and subjected to amino acid analysis. Quantification of amino acids was performed with the amino acid analyzer S 433 (Sykam, Fürstenfeldbruck, Germany). The injection volume was between 10 and 100 µL, and a PEEK column filled with the cation exchange resin LCA K07/Li (150 mm × 4.6 mm, 7 µM) was used for separation according to a custom gradient program. Loading and running buffers were purchased from Sykam. Post-column derivatization with ninhydrin was applied, followed by VIS detection with an integrated two-channel photometer at 440 and 570 nm. External calibration was performed with commercial amino acid standards (Sigma-Aldrich, Steinheim, Germany).

### Characterization of products

The ^1^H spectra of synthesized products were recorded with an Avance III HDX 500 MHz Ascend instrument (Bruker, Rheinstetten, Germany) at 500.13 MHz. For that, 5.0 mg of dry substance was dissolved in 650 µL of deuterium oxide. All chemical shifts are given in parts per million (ppm) relative to an internal HOD signal (^1^H; 4.70 ppm).

### Statistical treatment

All samples were analyzed at least in triplicate. Grubbs test was performed for outlier elimination. Normal distribution was tested with the Shapiro–Wilk test. Comparison of mean values was performed by one-way ANOVA. All statistical calculations were performed with the software Origin Pro 2019 (OriginLab).

### Synthesis of *α*-ketoacyl peptides via tosylation

#### Synthesis of pyruvoylglycine (8a)

Tosylation was performed according to a literature approach (Dixon 1964) with some modifications. Tosyl chloride (2.4377 g, 12.8 mmol) was dissolved in 6.25 mL acetone (dried over anhydrous sodium sulfate) and cooled down to 0 °C in an ice bath. Pyruvic acid (0.875 mL, 12.6 mmol) was slowly added to the solution with stirring, and then pyridine (1.025 mL, 12.7 mmol) was mixed in at 0 °C. The mixture was stirred for 20 min and afterwards, a solution of glycine (0.9375 g, 12.5 mmol) and pyridine (2.125 mL, 26 mmol) in water (8.75 mL) was added dropwise at 0 °C. The mixture was allowed to warm to room temperature and rapidly stirred until the previously formed precipitate had dissolved. The solution was reduced to dryness *in vacuo*. The residue was dissolved in 20 mL water, acidified with 1 mL 12 M hydrochloric acid and extracted with ethyl acetate (4 × 100 mL). The organic phases were combined, dried over sodium sulfate and evaporated to dryness *in vacuo*. The crude product was dissolved in 10 mL water, neutralized with 50 µL 5 M sodium hydroxide and chromatographed on a 1.5 × 50 cm glass column filled with 80 mL of strongly basic anion-exchange resin (AG 1-X8, BioRad Lab, Munich, Germany), previously regenerated and preconditioned by successive application of 300 mL 1 M sodium hydroxide, 600 mL water, 300 mL 1 M hydrochloric acid and washed neutral with 600 mL water. The elution was performed with 4 M acetic acid (500 mL). The fractions were collected in separate 5 mL tubes using a BioRad 2110 Fraction collector. Samples (50 µL) of each fraction were mixed with 50 µL of 0.1% (*m*/*v*) solution of DNPH in 2 M hydrochloric acid and incubated at room temperature for 5 min. The mixtures were then extracted with 250 µL ethyl acetate. The organic phases were separated and extracted with 350 µL 0.4 M sodium carbonate buffer, pH 9.6. Of each aqueous solution, 100 µL was transferred to a microtiter plate, and the absorbance at 365 nm was measured using the plate reader Tecan Infinite M200 Pro (Tecan Group, Männedorf, Switzerland) The fractions 51–67 (out of total 100) were tested positively (photometrically) and further characterized using HPLC–MS/MS. Fractions that contained the product were combined, dried *in vacuo* and lyophilized. The yield was 27.9 mg (0.192 mmol, molar yield = 3%).

#### Synthesis of pyruvoylglycylalanine (8b)

Tosylation was performed using the same approach as described above. For the reaction, 0.325 g (1.7 mmol) of tosyl chloride (in 0.833 mL dry acetone) was mixed with pyruvic acid (0.177 mL, 1.66 mmol) and pyridine (0.135 mL, 1.67 mmol) at 0 °C. After 20 min of stirring, a solution of glycylalanine (0.246 g, 1.68 mmol) and pyridine (0.283 mL, 3.6 mmol) in 1.167 mL water was added. After stirring, warming up and dissolving of the precipitate, the mixture was dried to syrup *in vacuo*, dissolved in 2 mL water and acidified with 100 µL of 12 M hydrochloric acid. The solution was then extracted with ethyl acetate (4 × 2.5 mL), and the combined organic phases were dried *in vacuo*. The residue was dissolved in 2 mL of water and neutralized with 2 µL of 5 M sodium hydroxide and chromatographically purified as described above. DNPH-positive fractions were identified as described above. The fractions 13–31 out of total 100 were tested positively (photometrically) and further characterized using HPLC–MS/MS. Fractions that contained the product were combined, dried *in vacuo* and lyophilized.

Analytical data: HPLC–ESI–MS/MS (*α*-KaP system): t_R_, 1.1 min; fragmentation, positive mode (100 V, 10 eV) of [M + H]^+^ (*m/z* 217): 44 (100), 90 (59), 100 (14), 30 (11); ^1^H-NMR (500 MHz, D_2_O), δ [ppm]: 1.37 (dd, 3H, *J* = 2.1, 9.1, Ala-CH_3_); 1.51 (s, 1.3 H, COCH_3_, hydrated form), 2.40 (s, 1.7 H, COCH_3_), 3.93 (d, 0.8H, J = 1.6 Hz, Gly-CH_2_, hydrated form); 3.98 (s, 1.2H, Gly-CH_2_), 4.33 (q, 1H, Ala-CH, *J* = 9.1). The yield was 4.2 mg (0.019 mmol, molar yield = 3%).

### Synthesis of *α*-ketoacyl peptides via metal-ion-catalyzed transamination

#### Synthesis of pyruvoylglycine (8a)

Transamination reactions were performed using modified literature approaches (Dixon 1964; Nishimura et al. 1998). Alanylglycine (0.100 g, 0.68 mmol) was dissolved in 50 mL of water. Then, a reagent solution was mixed containing glyoxylic acid monohydrate (3.75 g, 41 mmol), 1.2 mL of a 0.5 M cupric sulfate solution, and pyridine (1.5 mL, 7.4 mmol) filled up with water to 10 mL in total. The reagent solution was added to the peptide solution and the mixture stirred for 60 min at 25 °C. Reaction progress was verified by TLC and DNPH spraying reagent. To each synthesis mixture, 5 mL of 12 M hydrochloric acid was added. Acidified solutions were extracted with ethyl acetate (3 × 100 mL). The combined organic extracts were dried over sodium sulfate, filtrated and evaporated *in vacuo*. The residues were dissolved in 10 mL water and the pH was adjusted to 7 with 0.1 M acetic acid. The crude product solutions were chromatographed on a 1.5 × 50 cm glass column filled with 80 mL of strongly basic resin AG 1-X8 previously equilibrated as described above. The elution was conducted with 500 mL of each 1 M, 2 M, and 4 M acetic acid. Fractions of 5 mL were collected and target fractions were identified by TLC with DNPH spraying reagent. The positive fractions 110–150 were further characterized in scan and product ion scan measurements with HPLC–MS/MS. Combined fractions were dried *in vacuo*, the residue was dissolved in 10 mL of water and dried again until the product was free of acetic acid. Then the product was dissolved in 5 mL water and lyophilized.

Analytical data: Content by amino acid analysis was 56% based on glycine. Yield of pure substance was 53.8 mg (0.371 mmol, molar yield 54%).

#### Synthesis of glyoxylglycine (8c)

The synthesis was performed as described for pyruvoylglycine, but starting from glycylglycine (0.132 g, 1 mmol). The reagent solution consisted of glyoxylic acid monohydrate (0.75 g, 8 mmol), 2.4 mL of 0.5 M cupric sulfate solution and pyridine (1.5 mL, 7.4 mmol) filled up with water to 10 mL. After ion-exchange chromatography, the fractions 101–159 were shown to contain the target product by spotting test and HPLC–MS/MS. They were worked up as described above.

Analytical data: Content by amino acid analysis was 17% based on glycine. Yield of pure substance was 15.6 mg (0.119 mmol, molar yield 12%).

#### Synthesis of (3-methyl-2-oxo)valerylglycine (8e)

Prior to synthesis, the protecting group of Boc-IG-OH was removed. For that, Boc-IG-OH (0.306 g, 1.06 mmol) was dissolved in 3 mL THF. Then, 3 mL of 6 M hydrochloric acid was added and the mixture was stirred at room temperature for 3 h. The progress of removal of the Boc group was verified using TLC and detection with ninhydrin. After the complete removal of the Boc group, the solution was dried *in vacuo*, dissolved in 1 mL water and evaporated again. This process was repeated threefold until the product was free of hydrochloric acid. The crude dipeptide solution was lyophilized. Then, isoleucylglycine (0.189 g, 1 mmol) was dissolved in 10 mL of water, and the transamination procedure was conducted as described for pyruvoylglycine. The reagent solution was the same as for glyoxylglycine. After ion-exchange chromatography and characterization of fractions by spotting test and HPLC–MS/MS, the fractions 193–256 were combined and worked up as described above.

Analytical data: Content by amino acid analysis was 57% based on glycine. Yield of pure substance was 97.4 mg (0.512 mmol, molar yield 49%).

### Synthesis of *α*-ketoacyl peptides via reaction of peptides with 3,5-di-*tert*-butyl-*o*-benzoquinone (DTBBQ)

#### Synthesis of pyruvoylglycine (8a)

This set of transamination reactions was performed using a modified literature approach (Vinšova et al. 2005). Alanylglycine (0.290 g, 2 mmol) was dissolved in 20 mL of water, and a solution of DTBBQ (0.440 g, 2 mmol) in 40 mL ethanol was added at room temperature. Then, the reaction mixture was stirred at 50 °C for 30 min under nitrogen atmosphere. The reaction progress was monitored by TLC and further by HPLC–MS/MS. Then, the solution was evaporated to dryness *in vacuo* and purified by silica gel chromatography. The elution was conducted with 500 mL of each hexane, 1/1 (*v*/*v*) ethyl acetate in hexane, ethyl acetate, and finally 1/1 (*v*/*v*) ethyl acetate in methanol. Fractions of 10 mL were collected, and DNPH-positive fractions were combined and evaporated to dryness *in vacuo.* The residue was dissolved in 10 mL of water, and this solution was subjected to anion-exchange chromatography exactly as described above for metal-ion catalyzed transamination. The DNPH-positive fractions 120–140 were combined and lyophilized. The residue was dissolved in 20 mL of 50% (*v*/*v*) acetonitrile in water and further purified by semipreparative HPLC–UV on a Wellchrom high-pressure gradient system consisting of two pumps (K-1001), an online degasser, a UV-detector (K-2501), and a 16-port fractionation valve (K-16; all from Knauer). Fractionations were carried out using a stainless steel column (300 mm × 8 mm; Knauer) filled with Eurospher-100 RP-18 material (5 µM particle size) with a guard column (300 mm × 8 mm) filled with the same material. The injection volume was 2 mL, and the separation was performed at room temperature and at a flow rate of 1.4 mL/min. As mobile phases, the same eluents as for HPLC–UV of carbonyl compounds (see above) were used in a gradient program (0 min, 0% B; 10 min, 0% B; 35 min, 10% B; 70 min, 100% B; 75 min, 0% B; 80 min, 0% B). The absorbance was read at 214 nm. The eluates were collected at the retention time between 50 and 70 min, combined, lyophilized and dissolved in 10 mL of water. Afterwards, another purification by semipreparative HPLC was conducted under the same conditions using only water as the mobile phase. Product eluates were collected at the retention times between 6 and 10 min, combined and lyophilized.

Analytical data: HPLC–ESI–MS/MS (*α*-KaP system): t_R_, 1.0 min; fragmentation, positive mode (100 V, 10 eV) of [M + H]^+^ (*m/z* 146): 43 (100), 76 (54), 30 (31), 100 (9); ^1^H-NMR (500 MHz, D_2_O), δ [ppm]: 1.51 (s, 1.3H, Gly-CH_2_, hydrated form); 2.40 (s, 1.7H, Gly-CH_2_); 3.97 (s, 0.9 H, CH_3_, hydrated form); 4.02 (s, 1.1H, CH_3_). Content by amino acid analysis was 63% based on glycine, yield of pure substance was 130.8 mg (1.181 mmol, molar yield = 59%).

#### Synthesis of glyoxylglycine (8c)

This compound was synthesized as described above, but starting from glycylglycine (0.264 g, 2 mmol). After ion-exchange chromatography, DNPH-positive fractions 105–156 were combined and subjected to semipreparative HPLC.

Analytical data: HPLC–ESI–MS/MS (*α*-KaP system): t_R_, 0.9 min; fragmentation, positive mode (100 V, 10 eV) of [M + H]^+^ (*m/z* 132): 30 (100), 86 (95), 58 (31), 114 (5), 76 (2); ^1^H-NMR (500 MHz, D_2_O), δ [ppm]: 3.87 (s, 2H, CH_2_), 5.28 (s, 1H, CHO, hydrated form). Content by amino acid analysis was 69% based on glycine, yield of pure substance was 154.6 mg (1.181 mmol, molar yield = 59%).

#### Synthesis of glyoxylglycylglycine (8d)

This compound was synthesized as described above, but starting from glycylglycylglycine (0.378 g, 2 mmol). After ion-exchange chromatography, DNPH-positive fractions 57–120 were combined and subjected to semipreparative HPLC.

Analytical data: HPLC–ESI–MS/MS (*α*-KaP system): t_R_, 0.8 min; fragmentation, positive mode (100 V, 10 eV) of [M + H]^+^ (*m/z* 189): 79 (100), 30 (58), 86 (49), 143 (18), 114 (5), 58 (5); ^1^H-NMR (500 MHz, D_2_O), δ [ppm]: 3.97 (s, 2H, CH_2_); 3.99 (s, 2H, CH_2_), 5.31 (s, 1H, CHO, hydrated form). Content by amino acid analysis was 63% based on glycine, yield of pure substance was 57.3 mg (0.304 mmol, molar yield = 15%).

#### Synthesis of (3-methyl-2-oxo)-valerylglycine (8e)

This compound was synthesized as described above, but starting from isoleucylglycine (0.376 g, 2 mmol), which had previously been obtained from Boc-Ile-Gly by the procedure described above. After ion-exchange chromatography, DNPH-positive fractions 179–186 were combined and subjected to semipreparative HPLC.

Analytical data: HPLC–ESI–MS/MS (*α*-KaP system): t_R_, 5.9 min; fragmentation, positive mode (100 V, 10 eV) of [M + H]^+^ (*m/z* 188): 57 (100), 76 (11), 41 (10), 85 (2); ^1^H-NMR (500 MHz, D_2_O), δ [ppm]: 4.03 (s, 2H, Gly-CH_2_); 1.64 (m, 1H, Ile-CH), 1.38 (m, 2H, Ile-CH_2_), 1.04 (d, 3H, Ile-CH_3_, *J* = 7.1), 0.82 (t, 3H, Ile-CH_3_, *J* = 7.9). Content by amino acid analysis was 86% based on glycine, yield of pure substance was 291.5 mg (1.559 mmol, molar yield = 78%).

## Results and discussion

### Comparison of synthesis approaches

The synthesis approaches were compared based on the overall complexity of the process and the yield of *α*-ketoacyl peptides (Table 2). Tosylation couplings were successfully carried out, but the molar yields of products were lower than using other pathways. Furthermore, this reaction is only occurring in the absence of water, which is disadvantageous for synthesis of α-ketoacyl peptides with terminal glyoxylic acid, due to its availability only in hydrated form. The synthesis approach via transamination with DTBBQ (Vinšova et al. 2005) resulted in highest yields and purity for most products. Complexity of the synthesis and purification efforts were comparable to transamination with metal ions, leading to the conclusion that *α*-ketoacyl peptides are preferably synthesized using DTBBQ.

**Table 2:** Comparison of approaches for the synthesis of *α*-ketoacyl peptides

		Yield (pure substance) [mol%]		
		Tosylation (Dixon 1964)	Transamination (Nishimura et al. 1998)	Transamination (Vinšova et al. 2005)
Glyoxylglycine	(O)GG-OH	Not applicable	12	*59*
Pyruvoylglycine	(O)AG-OH	3	*54*	45
Pyruvoylglycylalanine	(O)AGA-OH	3	N/A	N/A
Glyoxylglycylglycine	(O)GGG-OH	N/A	N/A	*15*
(3-Methyl-2-oxo)-valerylglycine	(O)IG-OH	N/A	49	*78*

Data are given as molar yields (pure substance in mol%) of synthesized α-ketoacyl peptides. The most successful approach for each product is highlighted in italic

*N/A* not available

**Fig. 2:**
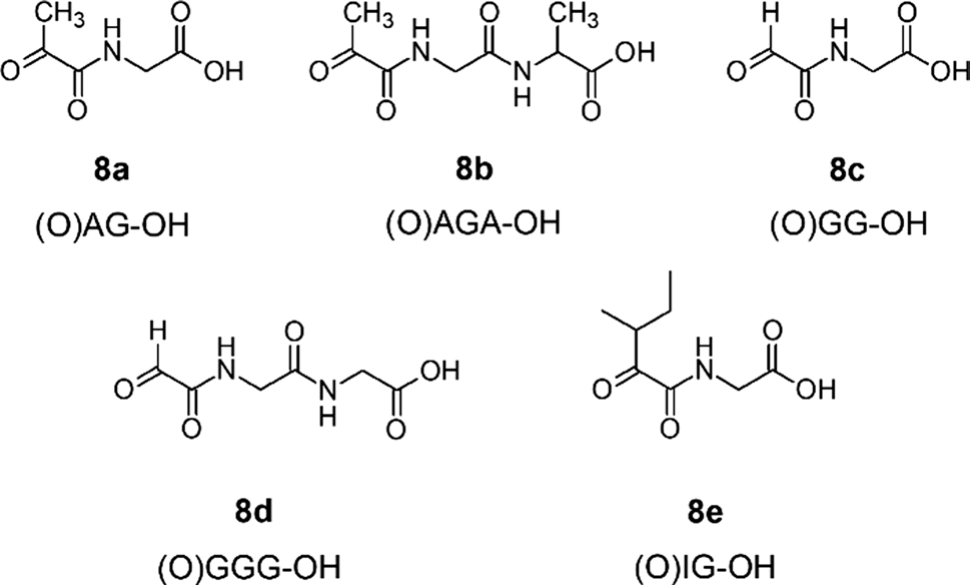
Structural formulas of pyruvoylglycine (8a, (O)AG-OH), pyruvoylglycylalanine (8b, (O)AGA-OH), glyoxylglycine (8c, (O)GG-OH), glyoxylglycylglycine (8d, (O)GGG-OH), and (3-methyl-2-oxo)-valerylglycine (8e, (O)IG-OH)

Differently from “normal” peptides, *α*-ketoacyl peptides have a carbonyl terminus, where an *α*-keto acid is bound to an amino acid or a peptide. To clearly denominate *α*-ketoacyl peptides and to distinguish them from the intact parent peptides while conserving the information about the side chain of the carbonylterminal *α*-keto acids, we propose that the sequence of the corresponding peptide is noted, but the hydrogen atom indicating the intact amino group is replaced by an “O” for oxygen written in brackets. For the *α*-ketoacyl peptide pyruvoylglycine (Fig. 2), which can formally be derived from alanylglycine (H-AG-OH), the notation would be (O)AG-OH.

### Study on the stability of *α*-ketoacyl peptides

The *α*-ketoacyl peptides formed during *α*-amidation of proteins are expected to be more reactive than regular peptides, since the *α*-carbonyl carbon atom is an electrophile and can react with nucleophiles such as water (Bell 1966). To examine the reactivity of *α*-ketoacyl peptides, incubations were carried out either in water, in 1% (*m*/*v*) acetic acid or as dry substance at room temperature, 100 °C and 150 °C as described above. Furthermore, we carried out a direct comparison of *α*-ketoacyl peptides and the corresponding unmodified peptides (with a terminal amino group) in regards of their stability and the occurrence of decomposition products.

**Fig. 3:**
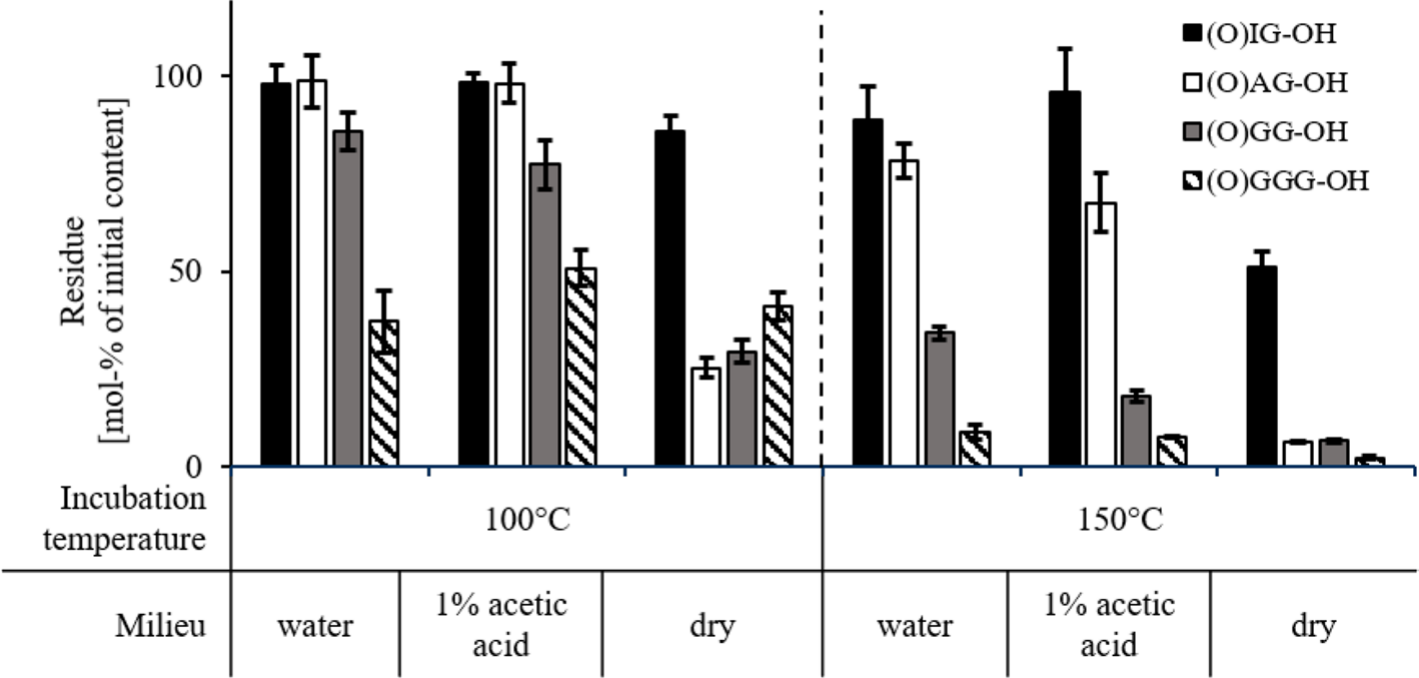
Residual content of (3-methyl-2-oxo)valerylglycine (black columns, (O)IG-OH), pyruvoylgycine (white columns, (O)AG-OH), glyoxylglycine (gray columns, (O)GG-OH) and glyoxylglycylglycine (hatched column, (O)GGG-OH) in mol% of initial substance after 2 h incubation at 100 °C and 150 °C. The incubations were carried out as 1 mM solutions in either water, 1% (*m*/*v*) acetic acid, or as dry substances. Data are mean values ± SD, *n* = 2–4

The examined *α*-KaPs (Fig. 2) showed a decreasing stability at higher temperatures. At room temperature, no significant decomposition of (O)GG-OH (8c) or (O)AG-OH (8a) was detected. However, the stability of examined substances decreased drastically at higher temperatures, as shown in Fig. 3. To maintain the comparability of the results only the residual contents of peptides after 2 h incubation were depicted, although it was observed that at high temperatures especially the dry substances decomposed very fast leading to up to 98% of the overall degradation in the first 30 min of incubations. The highest stability was demonstrated by (O)IG-OH (8e) only showing a significant decomposition as dry substance. (O)AG-OH (8a) proved to be stable in solution at 100 °C, while a significant decrease of concentration could be detected at 150 °C. The compound (O)GG-OH (8c) was less stable in solution even at 100 °C. The only structural difference between the examined *α*-ketoacyl dipeptides is the length of the acyl side chain at the carbonyl terminus relative to the overall size of the substances, so it can be safely assumed that the involvement of comparatively big side chains increases the stability of *α*-KaP. It was also observed that the compound (O)GGG-OH (8d) proved to be even less stable than (O)GG-OH (8c), which could be explained by generally higher reactivity of the triglycine chain compared to diglycine as described for unmodified compounds (Lu et al. 2005) and the ability to form cyclic glycylglycine as further degradation intermediate accelerating the overall decomposition of the *α*-KaP (Lee et al. 2015). The examined *α*-ketoacyl dipeptides are not able to undergo the cyclization reactions, which could explain their generally higher stability in comparison to (O)GGG-OH.

The decomposition process was further examined by analyzing the products formed in the process. The incubations of dry substances did not result in any significant increase in the content of carbonyl or amino components. One of the probable reaction pathways could be a pyrolysis followed by formation of azomethine ylides and their dimerization (Guerra and Yaylayan 2010) or further degradation to reactive amides, though this assumption must be further studied in the future.

**Fig. 4:**
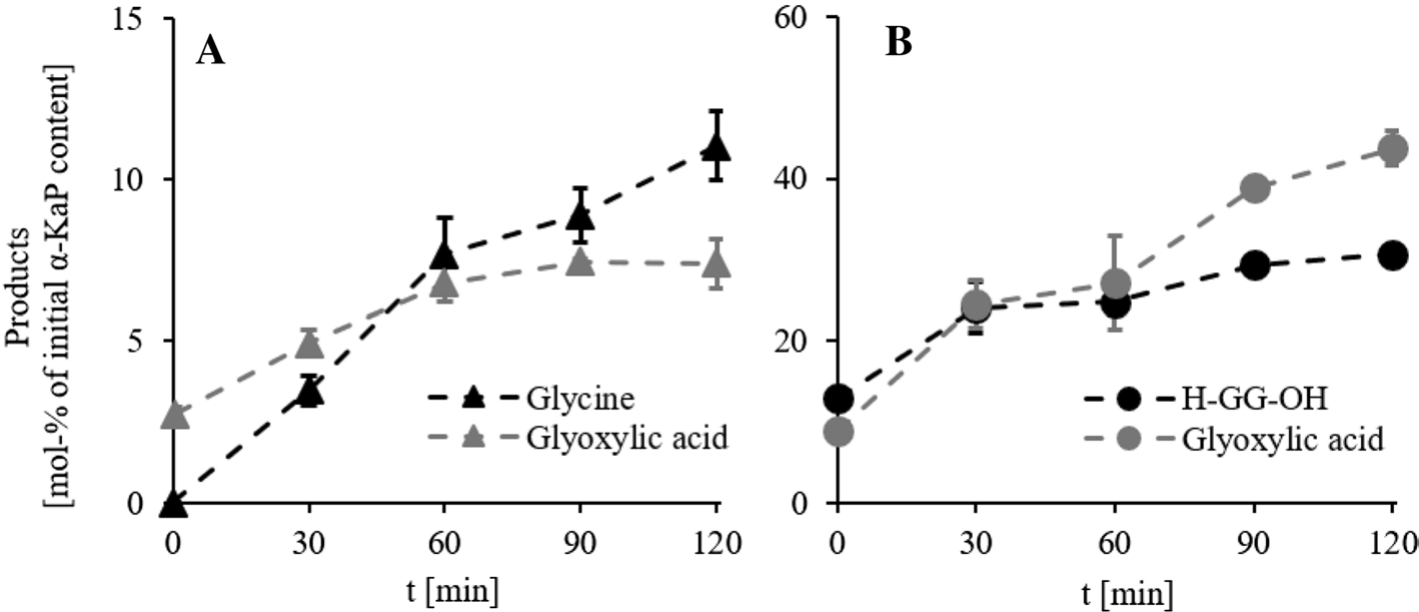
**a** Content of glycine (black triangles) and glyoxylic acid (gray triangles) formed as the result of hydrolysis of glyoxylglycine ((O)GG-OH) at 150 °C in water in mol% of initial *α*-KaP amount. **b** Content of glycylglycine (black circles, H-GG-OH) and glyoxylic acid (gray circles) formed as the result of hydrolysis of glyoxylglycylglycine ((O)GGG-OH) at 150 °C in water in mol% of initial substance. Data are mean values ± SD, *n* = 2–4

The decomposition of *α*-KaPs in the presence of water was proven to be represented by hydrolysis of the peptide bond resulting in the formation of an *α*-keto acid and an amino compound, as shown exemplarily for (O)GG-OH at 150 °C in water (Fig. 4a) by a significant increase in the content of glyoxylic acid and glycine. In addition, the assumption can be made, that the hydrolysis primarily takes place on the peptide bond closest to the *α*-keto group, since the decomposition of (O)GGG-OH predominantly leads to formation of glyoxylic acid and glycylglycine (e.g., reaction in water at 150 °C, Fig. 4b).

**Fig. 5:**
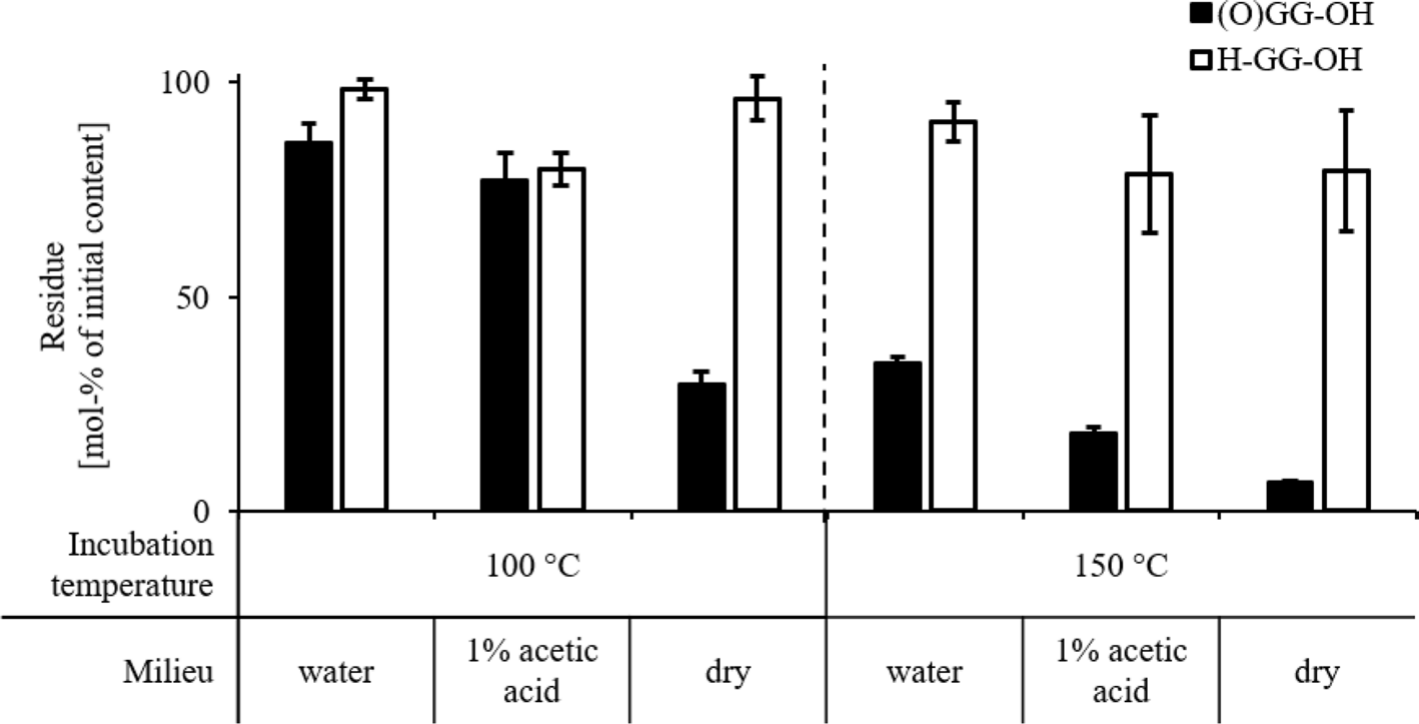
Residual content of glyoxylglycine (black columns, (O)GG-OH) and glycylglycine (white columns, H-GG-OH) in mol% of initial substance after 2 h incubation at room temperature, 100 °C and 150 °C in water, in 1% (*m*/*v*) acetic acid (1 mM peptide solutions) and as dry substance. Data are mean values ± SD, *n* = 2–4

Since the hydrolysis of regular peptides takes place only in strongly acidic (Roach and Gehrke 1970) or basic milieu (East 2018), direct comparisons of *α*-ketoacyl peptides and the corresponding regular peptides were carried out. At room temperature, no deviations could be verified after 48 h, since neither (O)GG-OH (8c) and (O)AG-OH (8a) nor the corresponding dipeptides glycylglycine (H-GG-OH) and alanylglycine (H-AG-OH) showed any significant decomposition. However, at higher temperatures, a significant difference between regular peptides and *α*-KaPs was observed (e.g., (O)GG-OH and H-GG-OH, Fig. 5). Glycylglycine showed higher stability under all examined conditions. The most significant differences between these two peptides were observed at 150 °C, when the regular peptide remained comparatively stable losing 10–22 mol% of its initial content, whereas up to 93 mol% of the *α*-ketoacyl peptide were decomposed. Other *α*-ketoacyl peptides and corresponding regular peptides were incubated under the same conditions showing similar trends. Since the *α*-keto-group is the only structural deviation of the corresponding peptides, it can be assumed that this group is able to provoke the decomposition of peptides.

### Proposed mechanism of peptide cleavage and catalytic role of the α-keto-group

Carbonyl groups are prone to nucleophilic addition, which can be slowed down by steric hindrance (Charton 1975) or by a positive inductive effect of neighboring groups (Taskinen and Kukkamäki 1977). The *α*-ketoacyl peptides examined in the present study demonstrate higher stability with increasing length of their alkyl side chains (Fig. 3), and consequently, with increasing occupied volume (Richards 1977) and inductive effect (Dwyer 2005) of those side chains. These findings lead to the assumption that the hydrolysis of the peptide bond nearest to the carbonyl terminus in *α*-KaP is initiated by a nucleophilic attack on the *α*-keto group, with the most probable reaction partner being water, since the hydrolysis could only be detected in aqueous solutions. Further evidence in favor of this hypothesis was obtained by ^1^H-NMR spectroscopy, showing a clear correlation between hydration rate and reactivity of the examined substances. The spectra of (O)GG-OH and (O)GGG-OH, the most reactive *α*-keto acyl peptides, only contained signals of respective geminal diols instead of aldehydes, proving a complete hydration similar to glyoxylic acid. On the contrary, (O)IG-OH remained in the keto-form, and no diol could be detected. The stability of (O)AG-OH was proven to be between that of (O)IG-OH and (O)GG-OH, and correspondingly, the signals of geminal diol and keto-form revealed ca. 40% of hydrated form for this α-ketoacyl peptide.

**Fig. 6:**
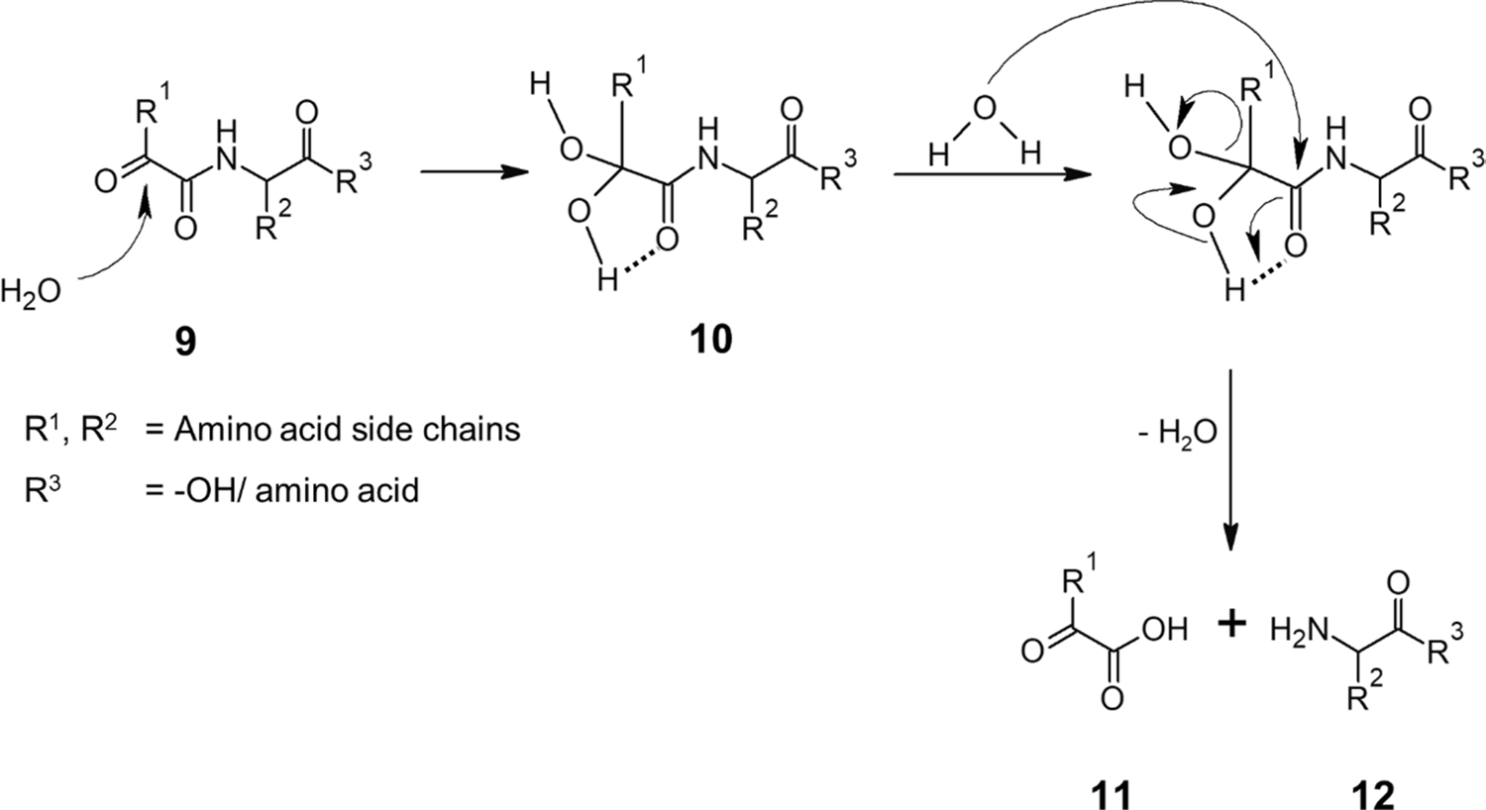
Proposed hydrolysis mechanism of *α*-ketoacyl peptides (9) with initial hydration to a geminal diol (10), subsequent cleavage of the peptide bond closest to the carbonyl terminus and formation of an *α*-keto acid (11) and an amino compound (12). R^1^ and R^2^ are amino acid side chains, R^3^ is -OH or amino acid

Based on these observations, we postulate the hydrolysis mechanism shown in Fig. 6. We assume a nucleophilic addition of water to *α*-ketoacyl peptides (**9**) resulting in a geminal diol (**10**). The hydroxyl group is expected to form a pseudo-pentagonal complex with the peptide oxygen atom in the process, interfering with the resonance stabilization of the amide bond. With subsequent application of energy, a further water molecule can attack the peptide carbon atom hence initiating the hydrolysis and leading to the formation of an *α*-keto acid (**11**) and an amino compound (**12**).

### Advanced degradation reactions

**Fig. 7:**
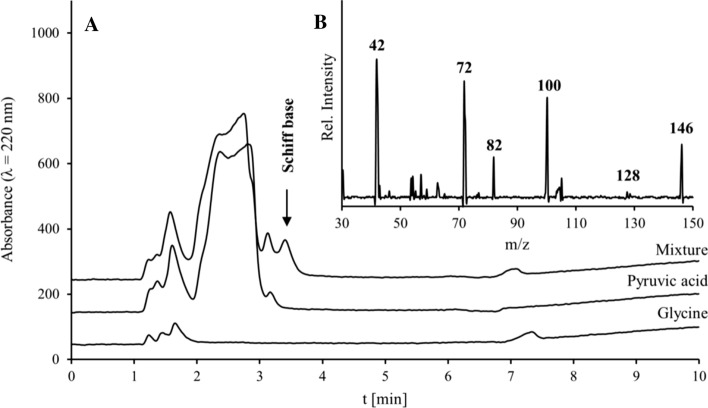
**a** HILIC chromatogram with UV and MS detections of glycine (10 mM), pyruvic acid (10 mM) and a mixture of both (each 10 mM) after 2 h incubation at 150 °C. **b** Mass spectrum of the peak at the retention time 3.4 min (Schiff base)

The *α*-keto acids released in hydrolysis of *α*-KaPs are electrophilic compounds, hence, they can react as well with water forming geminal diols (Sørensen et al. 1974; Griffiths and Socrates 1967). A further reaction partner can be the amino compound, so the formation of an imine (Schönberg and Moubacher 1952; Chu and Yaylayan 2008) can be expected. The plausibility of this assumption was verified through equimolar incubation of pyruvic and glyoxylic acids with glycine followed by a successful qualitative search for respective imines (Schiff bases) using HILIC-MS/MS, as exemplarily shown in Fig. 7 for a mixture after 2 h of incubation at 150 °C.

**Fig. 8:**
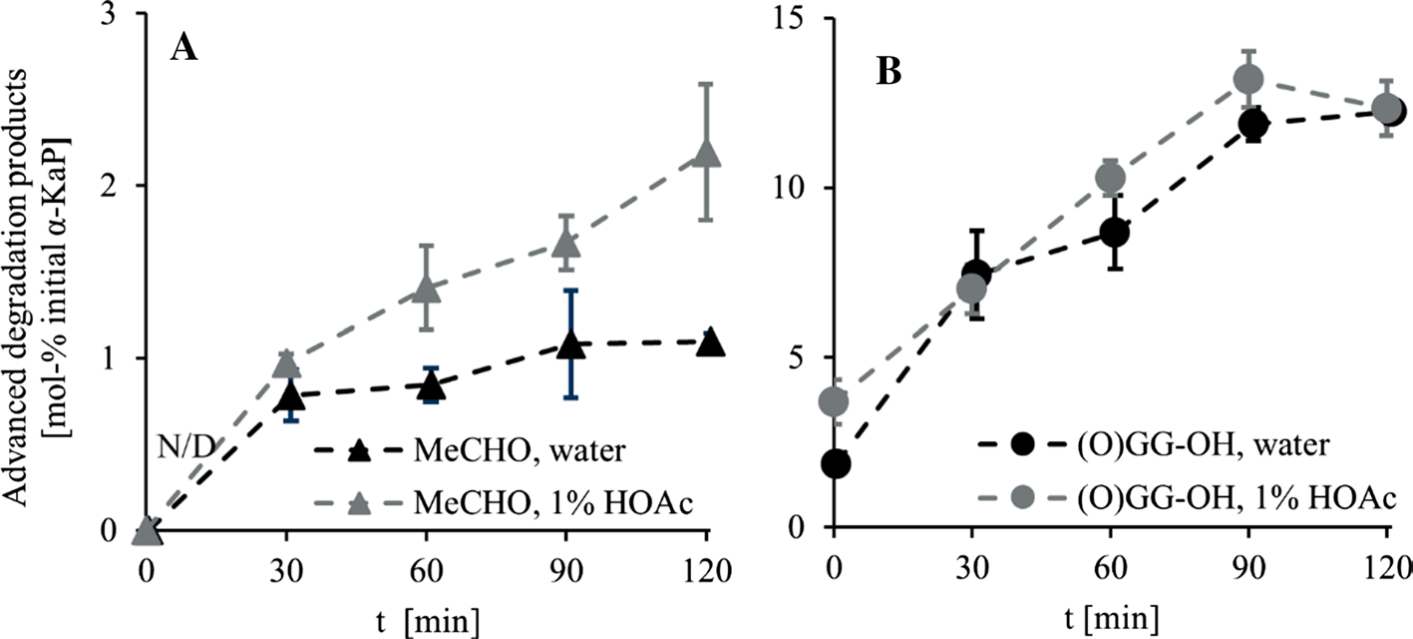
**a** Content of acetaldehyde (MeCHO) formed in advanced degradation reaction of 1 mM solutions of pyruvoylglycine ((O)AG-OH) at 150 °C in water (black triangles) and 1% (*m*/*v*) acetic acid (HOAc, gray triangles) in mol% of initial amount of *α*-KaP. **b** Content of glyoxylglycine ((O)GG-OH) formed in advanced degradation reaction of 1 mM solution of glyoxylglycylglycine ((O)GGG-OH) at 150 °C in water (black circles) and 1% (*m*/*v*) acetic acid (HOAc, gray circles). Data are mean values ± SD, *n* = 2–4

We were further able to detect a rise in concentration of Strecker aldehydes in the course of decomposition of *α-*ketoacyl peptides, e.g., acetaldehyde formed from (O)AG-OH (Fig. 8a). These substances can only originate from *α-*keto acids, previously emerged during hydrolysis. Since glyoxylic acid as well as pyruvic acid are not decomposed under the examined conditions, advanced degradation reactions are the only possible reason for the formation of aldehydes, the most likely being the Strecker-like decomposition of previously formed Schiff bases (Chu and Yaylayan, 2008) with subsequent decarboxylation of *α-*keto acids.

A closer look at the degradation of (O)GGG-OH further displayed a formation of (O)GG-OH, as shown in Fig. 8b. Glycylglycine (H-GG-OH) was stable at examined conditions, showing almost no oxidation, so no *α*-KaP could be formed in that pathway. Another possible reason could be the hydrolysis of the peptide bond closer to the C-terminus in (O)GGG-OH catalyzed by the *α-*keto group; however, we consider it unlikely owing to the long distance between both reaction partners. Therefore, we assume another nucleophilic reaction between initial hydrolysis products (glyoxylic acid and glycylglycine), leading to the formation of a Schiff base with subsequent hydrolysis resulting in a transamination (Hidalgo et al. 2013) of both reactants.

## Conclusion

**Fig. 9:**
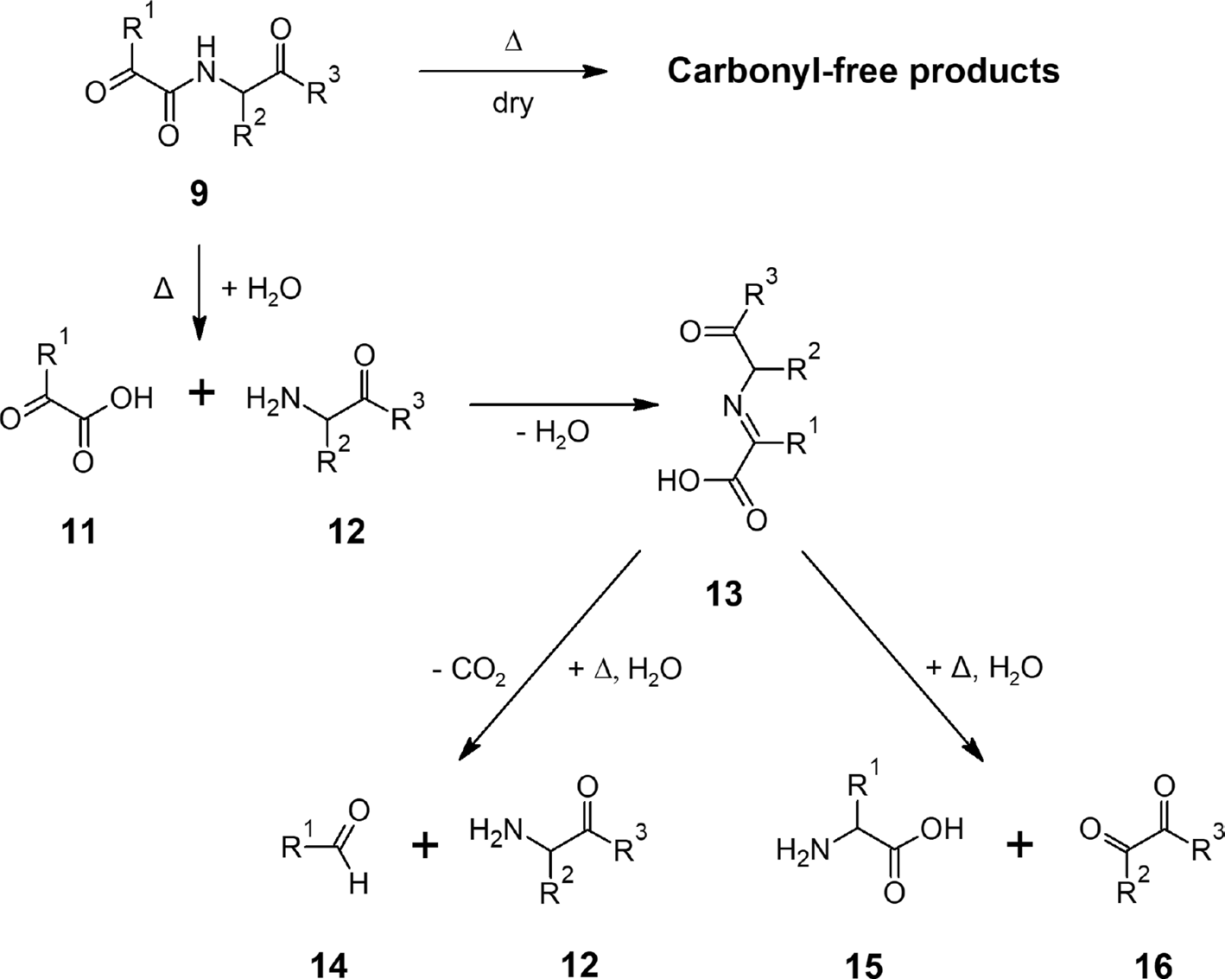
Proposed decomposition pathway of *α*-ketoacyl peptides (9) in the absence of water leading to carbonyl-free compounds and in the presence of water leading to hydrolysis into *α*-keto acid (11) and C-terminal amino compound (12), which can further react with each other forming a Schiff base (13). The latter can cleave and decarboxylate forming the Strecker aldehyde (14) of the *α*-keto acid or hydrolyze causing transamination and the formation of amino acid (15) and C-terminal *α*-keto compound (16). R^1^ and R^2^ are amino acid side chains, R^3^ is -OH or amino acid

In summary, optimal synthesis conditions for the preparation of α-ketoacyl peptides were identified. The studies on different *α*-ketoacyl peptides have confirmed our postulated hypothesis, that the *α*-keto group has a crucial influence on the reactivity and reaction pathways of *α*-ketoacyl peptides. We have determined a significant decline in stability of *α*-KaPs in comparison to their corresponding regular peptides resulting in higher hydrolysis rates. Furthermore, we have characterized and quantified the products of advanced degradation of examined modified peptides. Based on these observations, we propose the degradation pathway shown in Fig. 9. In the absence of water, the *α*-ketoacyl peptides (**9**) are expected to decompose into products without free carbonyl groups, but degradation into volatile compounds is also possible. These reactions will be subject of our further investigations. By application of energy, the prevalent reaction in the presence of water is a hydrolysis of the peptide bond next to the carbonyl terminus initiated by the hydration of the latter leading to the formation of an *α*-keto acid (**11**) and C-terminal amino compound (**12**). These are expected to react with each other and form a Schiff base (**13**), which can further be cleaved by decarboxylation of the previous *α*-keto acid, releasing a Strecker aldehyde (**14**). The hydrolytic cleavage of the Schiff base leads to transamination of intermediate compounds, forming the corresponding amino acid (**15**) and a new C-terminal *α*-keto compound (**16**).

Further approaches should be made to confirm and complete the proposed decomposition pathway, the most interesting being the carbonyl-free degradation products in absence of water. Degradation to various reactive intermediates should be confirmed and attempts for identification of products made. For more insights into formation of volatile degradation compounds, analysis with GC–MS/MS systems should be considered. Another important aspect is the influence of the acyl side chain on *α*-KaP reactivity in regards of steric inhibition and inductive effect. To further investigate the decomposition pathways, other environments such as the alkaline milieu or the presence of transition metals should be also considered.

## Abbreviations

*α*-KaP: *α*-Ketoacyl peptide
DNPH: 2,4-Dinitrophenylhydrazine
HPLC: High-pressure liquid chromatography
MS: Mass spectrometry
NFPA: Nonafluoropentanoic acid
(O)AG-OH: Pyruvoylglycine
(O)GG-OH: Glyoxylglycine
(O)GGG-OH: Glyoxylglycylglycine
(O)IG-OH: (3-Methyl-2-oxo)valerylglycine
ROS: Reactive oxygen species
RP: Reversed phase
TFA: Trifluoroacetic acid
THF: Tetrahydrofuran
TLC: Thin layer chromatography
